# Critical risks of haemoadsorption for COVID-19 patients and directions for future evaluations: a nationwide propensity score matched cohort study

**DOI:** 10.1038/s41598-025-13860-0

**Published:** 2025-08-09

**Authors:** Jan Andreas Kloka, Thomas Jasny, Oliver Old, Elina Nürenberg-Goloub, Christina Scharf, Patrick Meybohm, Alexander Supady, Kai Zacharowski, Benjamin Friedrichson

**Affiliations:** 1 Department of Anaesthesiology, Intensive Care Medicine and Pain Therapy, Goethe University, University Hospital Frankfurt, Frankfurt, Germany; 2 Department of Anaesthesiology, University Hospital, LMU Munich, Munich, Germany; 3 Department of Anaesthesiology, Intensive Care, Emergency and Pain Medicine, University Hospital Würzburg, Würzburg, Germany; 4 Interdisciplinary Medical Intensive Care, Faculty of Medicine, Medical Center - University of Freiburg, University of Freiburg, Freiburg, Germany; 5 Department of Anaesthesiology, Intensive Care Medicine and Pain Therapy, University Hospital Frankfurt, Theodor-Stern Kai 7, 60590 Frankfurt, Germany

**Keywords:** COVID-19, Intensive care unit, Haemoadsorption, In-hospital mortality, Cytokine adsorption, Hyperinflammation, Septic shock, Acute respiratory distress syndrome, Health care, Medical research, Epidemiology, Outcomes research, Risk factors

## Abstract

Haemoadsorption has been suggested as treatment adjunct for sepsis and septic shock, cardiac surgery, acute respiratory distress syndrome, and coronavirus disease 2019 (COVID-19). Randomised clinical trials did not provide conclusive evidence for benefits and even suggest risks in COVID-19 patients. Retrospective observational cohort study based on hospital remuneration data from all COVID-19 patients treated in intensive care units in Germany between 01/01/2020 and 12/31/2021. Regression modelling was performed for 1:1 propensity score matching of 2058 patients. Two-sided probability values for group comparisons and regression models with spline functions controlling for non-linear relationships and medically relevant interaction variables were calculated. In-hospital mortality of patients supported with haemoadsorption was significantly higher compared to matched control patients (74.6% vs. 70.3%, *p* = 0.0299). Haemoadsorption was associated with coagulopathy (68.0% vs. 54.9%, *p* < 0.0001), cardiac arrhythmia (49.2% vs. 44.2%, *p* = 0.0272), and cardiopulmonary resuscitation (CPR, 19.3% vs. 13.1%, *p* = 0.0002). Further, haemoadsorption increased the chance of death for COVID-19 patients without septic shock (odds ratio, OR [within a 95% confidence interval, CI]; 1.40 [1.05–1.86]) and did not improve survival of septic shock patients (1.19 [0.85–1.67]). Independent variables with a significant impact on mortality included the use of extracorporeal membrane oxygenation (ECMO, 2.15 [1.68–2.76]) and CPR (1.60 [1.03–2.45]). The timing of the haemoadsorption therapy had no effect on patients´ outcomes. Due to inconclusive evidence for benefit and potential harm, haemoadsorption therapy should be limited to thoroughly designed clinical trials before introduced into clinical routine in the context of COVID-19.

**Supplementary Information:**

The online version contains supplementary material available at 10.1038/s41598-025-13860-0.

## Introduction

Extracorporeal haemoadsorption was primarily introduced as an adjunctive treatment option for sepsis^1^ and is increasingly being used for critically ill patients with conditions related to hyperinflammatory states^2^, such as, septic shock, acute respiratory distress syndrome (ARDS), and severe coronavirus disease 2019 (COVID-19)^1–5^. The rationale for its use is the elimination of excess inflammatory mediators from the bloodstream^1,2^. Haemoadsorption can be combined with haemodialysis, haemofiltration or extracorporeal membrane oxygenation (ECMO), or it can be implemented as a stand-alone treatment^6^. The adsorption of molecules is unspecific. Pre-clinical and clinical studies have shown effective adsorption of pro-inflammatory cytokines and also anti-inflammatory mediators like interleukin (IL)-10 and granulocyte colony-stimulating factor (G-CSF)^7,8^. In addition, haemoadsorption was demonstrated to significantly remove of antiinfectives, anticoagulants, and antiarrythmics from the blood^9^.

In critical COVID-19 patients, haemoadsorption, among other extracorporeal blood purification techniques, was suggested for the mitigation of severe inflammatory states^10^. Increased levels of pro-inflammatory cytokines were shown in certain patient groups during the course of COVID-19 and attempts to counteract their harmful effects have emerged early on during the pandemic^11^. However, a number of randomised clinical trials (RCTs) with immunomodulatory drugs have not led to a clear therapeutic strategy beyond the indication for corticosteroids, IL-6 receptor antagonists, or Janus kinase (JAK)-inhibitors in defined patient groups^12,13^. Several independent studies challenge the assumption that hypercytokinaemia is the main cause of COVID-19 deaths in ICUs and emphasise the complex role of the inflammatory response in both the clinical manifestation and treatment of the disease^14,15^.

Early experience during the COVID-19 pandemic suggested haemoadsorption as an effective means for the reduction of pro-inflammatory mediators including IL-6 and associated clinical benefits for patients with severe presentations of COVID-19^8,16–18^. Previous analysis of the experience with septic patients showed that early initiation of haemoadsorption (≤ 12 h vs. > 24 h from the onset of septic shock) was critical for survival^19^. The “CytoSorb Therapy for COVID-19 (CTC)” registry-based study of 100 retrospectively selected patients with COVID-19-related ARDS supported with ECMO postulated that early (≤ 87 h after initiation of ECMO) implementation of haemoadsorption with the CytoSorb device (CytoSorbents Europe, Berlin, Germany) was associated with positive effects on the duration of organ support, intensive care, and 90-day mortality^20^. However, these analyses have to be considered with caution, since the study lacks adequate control groups, is biased with respect to the selection of patients and analytical parameters, and does not inform on important therapeutic variables^21^.

Three randomised clinical trials have evaluated the use of haemoadsorption with the CytoSorb® device in critically ill COVID-19 patients. In these studies, survival was not improved and a reduction of inflammatory markers was not conclusively shown for patients with haemoadsorption^22–24^. Also, the generalisability of these preliminary findings is limited due to single-centre design and small numbers of patients included in these trials (*n* < 50).

Despite the widespread use of haemoadsorption and initial promising reports, RCTs have not definitively confirmed its benefits for COVID-19 patients. For one thing, COVID-19 is still a poorly understood disease and the role of the immune response in its clinical manifestation has not been clarified. Secondly, the method of haemoadsorption has not been fully characterised in clinical application and the factors that are decisive for the success of the therapy are not entirely known. The aim of this study is to add additional evidence from a very large nation-wide cohort of COVID-19 patients treated with and without haemoadsorption in German ICUs during the pandemic.

## Methods

### Study design, study population, and data source

We conducted a nation-wide, retrospective, observational cohort study. All patients aged 18 years and older with PCR-confirmed SARS-CoV-2 infection (U07.1) treated in an ICU in Germany between 01/01/2020–12/31/2021 were included. We used hospital remuneration data from the German Federal Statistical Office (Statistisches Bundesamt, DESTATIS; https://www.destatis.de/DE/Home/_inhalt.html). Haemoadsorption patients were selected according to the operation and procedure code (*Operationen- und Prozedurenschlüssel*, OPS) for “extracorporeal adsorption of low- and medium molecular weight hydrophobic substances (including cytokine adsorption)”; 8-821.2. Our dataset does not provide indications and medical rationales for the use of haemoadsorption, therefore these are not considered in our analysis.

### Procedures and outcomes

According to the hospital financing act, all hospitals in Germany are obliged to report diagnosis-related groups (DRG) - coded diagnoses and OPS for reimbursement purposes for all patients to the health insurance funds and to the Institute for the Hospital Remuneration System (*Institut für das Entgeltsystem im Krankenhaus*, InEK) (*Krankenhausfinanzierungsgesetz*, KHG). The basis for the DRG system includes the implementation of the 10th version of the International Statistical Classification of Diseases and Related Health Problems (ICD10), which has been used to request the data (supplementary Tables 1–3). The data are anonymised and locally stored at a patient level at DESTATIS. The authors had no direct access to the database and conducted the queries and calculations remotely on the DESTATIS servers. The results were transmitted in an aggregated version. For data privacy reasons, variables expressed by less than three patients were not provided. Due to the retrospective nature of the study and the institutionally ensured anonymization, the Ethics Committee of University Hospital Frankfurt waived the requirement for ethical approval and the need to obtain informed consent (Ref: 2022–766). In our analysis, the primary endpoint was in-hospital mortality. The secondary endpoints included the hospital length-of-stay and the rate of major complications (coagulopathy, CPR, intracranial bleeding, stroke, pulmonary embolism, cardiac arrhythmia, embolism and/or thrombosis, myocardial Infarction, anoxic brain damage, cerebral edema, acute renal failure). We also analysed the timing and dosage of haemoadsorption therapy with regard to the primary endpoint.

### Statistical analysis

**Fig. 1:**
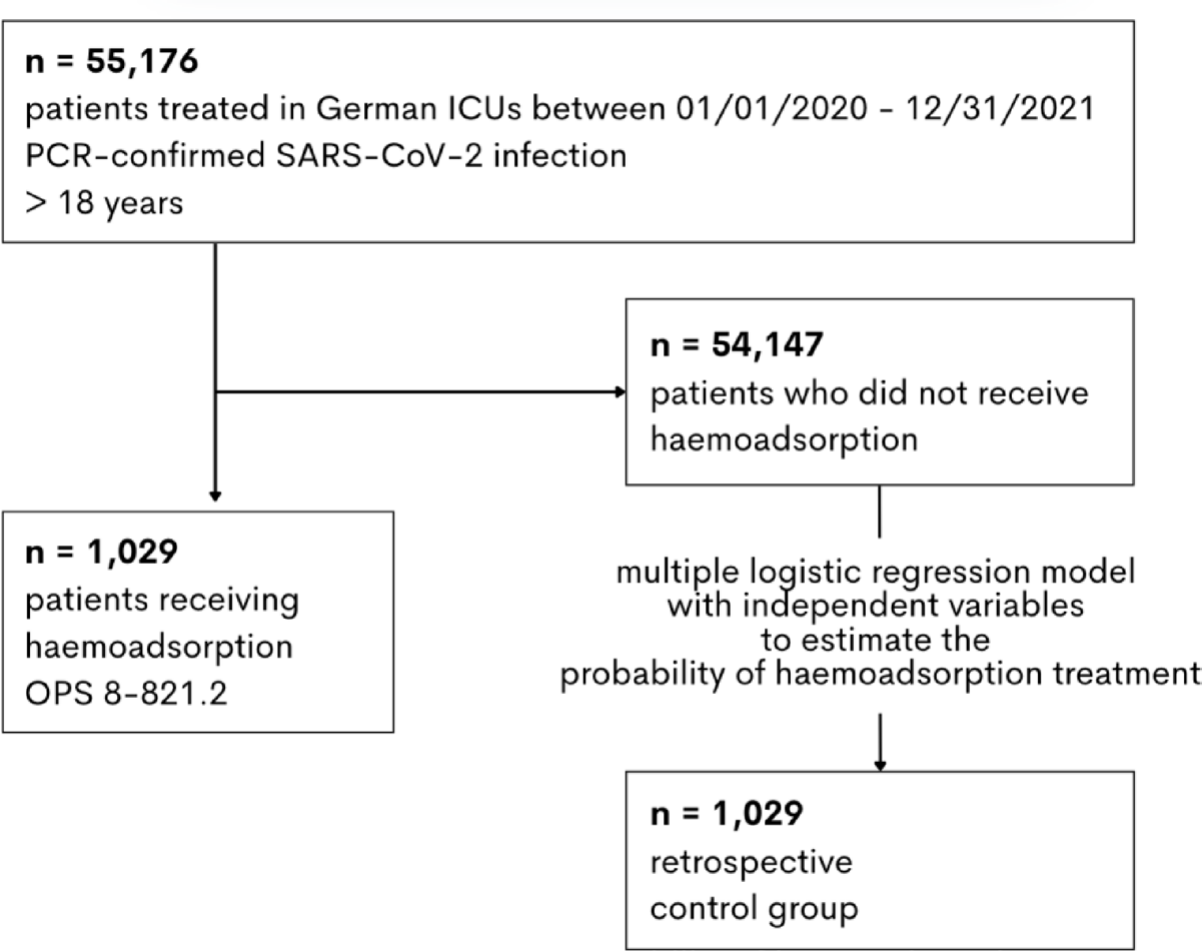
Flowchart for patient inclusion in the haemoadsorption and control groups. Each patient receiving haemoadsorption was paired with a control patient who had the most similar propensity score, representing the estimated probability of receiving haemoadsorption based on independent variables.

Data were analysed both descriptively and inferentially. Categorical variables are presented as absolute numbers and percentages, while non-normally distributed continuous variables are expressed as medians with interquartile ranges (25th and 75th percentiles). To assess the patients’ health state, we calculated the Elixhauser comorbidity score (EH-Score), which comprises the weighted analysis of 30 comorbidity groups. It has been validated with the use of administrative data within the ICD-10 system for in-hospital death as outcome and was shown to be superior to comparable scoring approaches^25^. We performed a 1:1 propensity score matching^26^ (k nearest neighbour^27^, based on the probability of the patients to receive haemoadsorption) to construct a robust retrospective control group consisting of 1029 patients who did not receive haemoadsorption. To estimate the probability of haemoadsorption, we utilised a multiple logistic regression model with independent variables, including the binary variables sex, ECMO and dialysis treatment and various complications and comorbidities (septic shock, acute and chronic liver failure, congestive heart failure, hypertension, chronic pulmonary disease, diabetes, renal and malignant diseases, obesity, and circulatory arrest prior to hospitalisation). Age was considered as continuous variable and modelled as a cubic B-Spline function with three inner knots distributed according to quantiles to account for nonlinearity. The selection of comorbidities and complications was based on medical relevance, availability in the dataset, and modelling considerations. Notably, these comorbidities and complications are not part of the Elixhauser score (Fig. 1).

To analyse the effect of therapy timing (from ICU admission to haemoadsorption deployment) and the number of haemoadsorption treatments (single vs. multiple cartridges used), we conducted a multiple logistic regression model based on the haemoadsorption population (*n* = 1029 patients) to estimate the probability of in-hospital mortality (included additional variables: gender, EH-Score, ECMO and dialysis treatment, septic shock, stroke, pulmonary embolism, embolism and/or thrombosis, myocardial infraction, CPR and circulatory arrest prior to hospitalisation). Timing of therapy onset was considered as a continuous variable and modelled as a cubic B-Spline function with four inner knots distributed according to quantiles to account for nonlinearity. The final selection of variables for our model was based on the Akaike Information Criterion (AIC)^28^.

Static group differences were assessed using the Chi-square test for binary variables and the Wilcoxon-rank sum test for continuous variables^29,30^, as none of the considered variables followed a normal distribution. All tests were two-tailed, with a significance level set at 5%. All analyses were conducted using SAS (Version 9.4M6, SAS Institute Inc., Cary, NC, USA), Excel 2019 (Microsoft Corp., Seattle, WA, USA) and the Python Pandas library for data manipulation (version: 2.1.3), NumPy for numerical computations (version: 1.26.2), and SciPy statistical functions (version: 1.11.4), specifically the chi2_contingency method from the scipy.stats module, without Yates correction.

## Results

During the 2-year study period in 2020–2021, 55,176 COVID-19 patients were treated in 340 German ICUs and 1029 (1.9%) received haemoadsorption in 204 ICUs. Using 1:1 propensity score matching, we successfully constructed a retrospective control cohort of 1029 patients that did not receive haemoadsorption (Fig. 1).

Most patients (470/1029 [45.7%]) received one application (equivalent to one haemoadsorption cartridge), 207/1029 patients (20.1%) received two applications and 352/1029 patients (34.2%) were treated with three or more applications of haemoadsorption. Patient characteristics are displayed in Table 1, including the propensity-matched cohort without haemoadsorption. 768/1029 patients (74.6%) in the haemoadsorption group died in hospital compared to 723/1029 patients (70.3%) in the propensity-matched control group, revealing haemoadsorption as a significant risk factor for mortality (*p* = 0.0299). Gender (810/1029 [78.7%] males in the haemoadsorption group versus 838/1029 [81.4%] males in the control group, *p* = 0.1362) and age (median of 62 years within an interquartile range [IQR] of 54–69 years vs. 62 [55–69] years, *p* = 0.4995) and comorbidities as assessed by the Elixhauser score (15 [9-23] vs. 15 [8-22], *p* = 0.0683) were balanced between the two groups. Haemoadsorption patients had longer hospital stays (22 [13-39] vs. 21 [12-37] days, *p* = 0.0401). In the haemoadsorption group, patients were prone to coagulopathies (68.0% vs. 54.9% in the control group, *p* < 0.0001) and cardiac arrythmia (49.2% vs. 44.2%, *p* = 0.0272), while pulmonary embolism (11.6% vs. 15.1%, *p* = 0.0231) and stroke (2.2% vs. 4.3%, *p* = 0.0130) were observed less frequently. CPR was significantly more abundant in the haemoadsorption group (19.3% vs. 13.1%, *p* = 0.0002) but only 10.5% of the haemoadsorption patients suffered in-hospital cardiac arrest (IHCA) prior to the haemoadsorption treatment, which strengthens the matching in this respect. The use of ECMO and renal replacement therapy were matching factors and therefore balanced between the two groups.

**Table 1:** Outcomes and major characteristics of the haemoadsorption group and the propensity score matched control group. The* p* values indicate the statistical impact on haemoadsorption deployment.

	Haemoadsorption group (*n* = 1029)		Control group (*n* = 1029)		
*Primary outcome*					*p*
In-hospital deaths	768 (74.64%)		723 (70.26%)		0.0299
*Secondary outcomes*					
Hospital length-of-stay, median (IQR)	22 (13–39) days		21 (12–37) days		0.0401
Coagulopathy	700	(68.03%)	565	(54.91%)	< 0.0001
CPR	199	(19.34%)	135	(13.12%)	0.0002
Intracranial Bleeding	88	(8.55%)	106	(10.30%)	0.1997
Stroke	23	(2.24%)	44	(4.28%)	0.0130
Pulmonary embolism	119	(11.56%)	155	(15.06%)	0.0231
Cardiac arrhythmia	506	(49.17%)	455	(44.22%)	0.0272
Embolism and/or thrombosis	24	(2.33%)	30	(2.92%)	0.4905
Myocardial Infarction	28	(2.72%)	34	(3.30%)	0.5191
Anoxic brain damage	15	(1.46%)	13	(1.26%)	0.8491
Cerebral edema	31	(3.01%)	34	(3.30%)	0.8019
Acute renal failure	894	(86.88%)	887	(86.20%)	0.6984
*Propensity score variables (matching characteristics)*					
Age, median (IQR)	62 (54–69) years		62 (55–69) years		0.4995
Gender (Male)	810	(78.72%)	838	(81.44%)	0.1362
ECMO	464	(45.09%)	463	(45.00%)	1.0000
Dialysis	975	(94.75%)	975	(94.75%)	1.0000
Septic shock	519	(50.44%)	535	(51.99%)	0.5083
Circulatory arrest prior to hospitalisation	12	(1.17%)	13	(1.26%)	1.0000
Congestive heart failure	262	(25.46%)	240	(23.32%)	0.2811
Hypertension	569	(55.30%)	551	(53.55%)	0.4518
Chronic pulmonary disease	138	(13.41%)	121	(11.76%)	0.2876
Chronic liver disease (w/o acute liver failure)	74	(7.19%)	71	(6.90%)	0.8632
Diabetes	358	(34.79%)	335	(32.56%)	0.3048
Renal disease (w/o acute renal failure)	194	(18.85%)	183	(17.78%)	0.5688
Malignant diseases	34	(3.30%)	26	(2.53%)	0.3591
Acute liver failure	339	(32.94%)	306	(29.74%)	0.1284
Obesity	182	(17.69%)	169	(16.42%)	0.4819
*Additional characteristics (not matched)*					
VV-ECMO^#^	443	(43.05%)	447	(43.44%)	0.8938
VA-ECMO^#^	34	(3.30%)	27	(2.62%)	0.4355
IHCA prior to haemoadsorption treatment	108	(10.50%)	nr.	nr.	
Elixhauser score, median (IQR)	15 (9–23)		15 (8–22)		0.0683

Data are n (%), unless otherwise stated. w/o, without; IQR, interquartile range; ECMO, extracorporeal membrane oxygenation (all types); VV, veno-venous; VA, veno-arterial; CPR, cardiopulmonary resuscitation; IHCA, in-hospital cardiac arrest; nr. = not relevant; ^#^patients were matched according to any ECMO treatment but not specifically VV or VA-ECMO; which did not differ significantly between the groups supporting the matching quality.

**Fig. 2:**
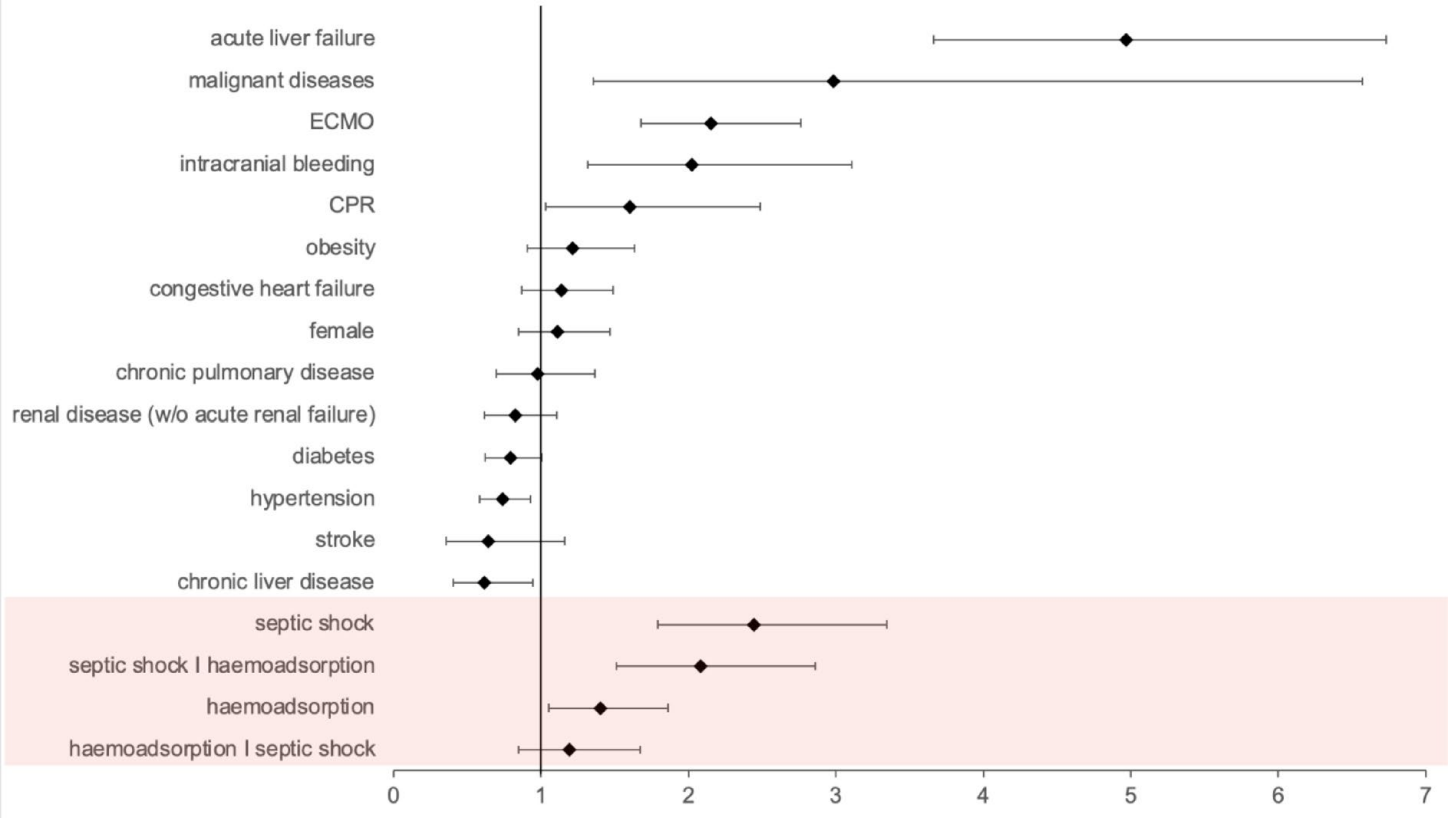
Haemoadsorption is a significant risk factor for death in the multiple logistic regression model. Obesity, congestive heart failure, gender, chronic pulmonary disease, renal disease, diabetes, and stroke were statistically insignificant parameters for mortality within the study group but were included in the model due to AIC improvement. Interaction variables show OR for death from (i) septic shock in the control group (septic shock), (ii) Septic shock in the haemoadsorption group (septic shock I haemoadsorption), (iii) Haemoadsorption in the non-septic shock group (haemoadsorption), and (iv) Haemoadsorption in the septic shock group (haemoadsorption I septic shock). ECMO = extracorporeal membrane oxygenation, CPR = cardiopulmonary resuscitation, w/o = without.

We adjusted a multiple logistic regression for our study population (*n* = 2058) based on the matching variables (Fig. 2). The probability of mortality was dependent on acute liver failure (odds ratio [OR] 4.97 within a 95% confidence interval [CI] of 3.66–6.73), malignant disease (2.99 [1.36–6.57]), intracranial bleeding (2.02 [1.32–3.11]), the use of ECMO (2.15 [1.68–2.76]) and CPR (1.60 [1.03–2.49]). We also controlled for gender, obesity, congestive heart failure, chronic pulmonary disease, renal disease, diabetes, hypertension, stroke, and chronic liver disease which increased the model’s relevance in terms of AIC. To account for the interaction between haemoadsorption treatment and septic shock, we included an interaction variable (Fig. 2). Haemoadsorption significantly increased the risk of in-hospital death in COVID-19 patients without a septic shock (Fig. 2, haemoadsorption: 1.40 [1.05–1.86]). Survival was not improved by haemoadsorption in patients with septic shock (haemoadsorption I septic shock: 1.19 [0.85–1.67]). Conversely, septic shock was detrimental in both groups (septic shock: 2.45 [1.79–3.34] and septic shock I haemoadsorption: 2.08 [1.51–2.86]). Multiple logistic regression analysis thus confirms the results of the group comparisons, suggesting haemoadsorption, when used in critically ill COVID-19 patients, is associated with higher mortality.

We sought to determine variables associated with the mortality of patients receiving haemoadsorption (Table 2). Non-survivors (*n* = 768) were significantly older (median age of 63 [IQR 56–70] years) compared to the survivors (*n* = 261, 59 [56–64] years, *p* < 0.0001), had a weaker health state resulting in a higher Elixhauser score of 16 (10–24) compared to 12 (6–20) in the survivor group (*p* < 0.0001), and shorter hospital stays of 16 (11–30) days compared to the survivors (39 [26–64], *p* < 0.0001). Even though male patients were overrepresented in both groups (Table 1), the gender had no significant impact on survival. Fatalities were statistically associated with the occurrence of septic shock (35.6% of the survivors vs. 55.5% of the non-survivors, *p* < 0.0001), acute liver failure (12.6% vs. 39.6%, *p* < 0.0001), and malignant diseases (1.2% vs. 4.0%, *p* = 0.0242). ECMO support was initiated for 94 (36.0%) survivors compared to 370 (48.2%) patients in the non-survivor group with a significant impact on mortality (*p* = 0.0006). Acute renal failure showed no statistically significant correlation with mortality, however, the initiation of dialysis was detrimental (92.3% vs. 95.6%, *p* = 0.0428) as was CPR (14.2% vs. 21.1%, *p* = 0.0145). Although coagulopathies and cardiac arrythmia occurred significantly more often while stroke and pulmonary embolism were less common in the haemoadsorption group compared to the control group (Table 1), none of these complications and comorbidities had an impact on the survival of haemoadsorption patients (Table 2). In contrast, mortality among the patients in the control group was associated with coagulation disorders including coagulopathy (46.1% of the survivors vs. 58.6% of the non-survivors, *p* = 0.0002), pulmonary embolism (11.4% vs. 16.6%, *p* = 0.0344), intracranial bleeding (4.6% vs. 12.7%, *p* = 0.0001), and myocardial infarction (1.3% vs. 4.2%, *p* = 0.0197). Other factors that significantly correlated with mortality in the control group were overlapping with the haemoadsorption group and included older age (median of 60 [IQR 52–67] years for survivors vs. 63 [56–70] years for non-survivors, *p* < 0.0001), a higher Elixhauser score (5 [13-19] vs. 9 [16-23], *p* < 0.0001), shorter hospital stays (39 [21–57] days vs. 17 [10-28] days, *p* < 0.0001), acute liver failure (10.1% vs. 38.0%, *p* < 0.0001), septic shock (33.3% vs. 59.9%, *p* < 0.0001), the use of ECMO (37.2% vs. 48.3%, *p* = 0.012), CPR (8.2% vs. 15.2%, *p* = 0.0022), and dialysis (90.2% vs. 96.7%, *p* < 0.0001). Fatalities in the control group were additionally associated with acute renal failure (75.8% vs. 90.6%, *p* < 0.0001) but in contrast to the haemoadsorption group, patients with renal disease had better outcomes (23.9% vs. 15.2%, *p* = 0.0009) and malignant disease had no impact on mortality (2.0% vs. 2.8%, *p* = 0.4517).

**Table 2:** Major characteristics of the survivors and non-survivors in the haemoadsorption group. The* p* values indicate the statistical impact on survival.

	Survivors (*n* = 261)		Non-survivors (*n* = 768)		
*Secondary outcomes*					*p*
Hospital length-of-stay, median (IQR)	39 (26–64) days		19 (11–30) days		< 0.0001
Coagulopathy	166	(63.60%)	534	(69.53%)	0.0760
CPR	37	(14.18%)	162	(21.09%)	0.0145
Intracranial Bleeding	20	(7.66%)	68	(8.85%)	0.5521
Stroke	8	(3.07%)	15	(1.95%)	0.2938
Pulmonary embolism	24	(9.20%)	95	(12.37%)	0.1659
Cardiac arrhythmia	117	(44.83%)	389	(50.65%)	0.1040
Embolism and/or thrombosis	3	(1.15%)	21	(2.73%)	0.1427
Myocardial Infarction	4	(1.53%)	24	(3.13%)	0.1719
Anoxic brain damage	4	(1.53%)	11	(1.43%)	0.9070
Cerebral edema	4	(1.53%)	27	(3.52%)	0.1054
Acute renal failure	222	(85.06%)	672	(87.50%)	0.3126
*Propensity score variables*					
Age, median (IQR)	59 (56–64) years		63 (56–70) years		< 0.0001
Gender (Male)	211	(80.84%)	599	(77.99%)	0.3315
ECMO	94	(36.02%)	370	(48.18%)	0.0006
Dialysis	241	(92.34%)	734	(95.57%)	0.0428
Septic shock	93	(35.63%)	426	(55.47%)	< 0.0001
Circulatory arrest prior to hospitalisation	4	(1.53%)	8	(1.04%)	0.5233
Congestive heart failure	57	(21.84%)	205	(26.69%)	0.1199
Hypertension	156	(59.77%)	413	(53.78%)	0.0924
Chronic pulmonary disease	34	(13.03%)	104	(13.54%)	0.8330
Chronic liver disease (w/o acute liver failure)	22	(8.43%)	52	(6.77%)	0.3703
Diabetes	99	(37.93%)	259	(33.72%)	0.2177
Renal disease (w/o acute renal failure)	43	(16.48%)	151	(19.66%)	0.2555
Malignant diseases	3	(1.15%)	31	(4.04%)	0.0242
Acute liver failure	33	(12.64%)	306	(39.84%)	< 0.0001
Obesity	50	(19.16%)	132	(17.19%)	0.4712
*Additional characteristics*					
VV-ECMO^#^	86	(32.95%)	357	(46.48%)	0.0001
VA-ECMO^#^	10	(3.83%)	24	(3.13%)	0.5812
IHCA prior to haemoadsorption treatment	20	(7.66%)	88	(11.46%)	0.0839
Elixhauser score, median (IQR)	12 (6–20)		16 (10–24)		< 0.0001

Data are n (%), unless otherwise stated. w/o, without, IQR, interquartile range; ECMO, extracorporeal membrane oxygenation; VV, veno-venous; VA, veno-arterial; CPR, cardiopulmonary resuscitation; IHCA, in-hospital cardiac arrest; ^#^patients were matched according to any ECMO treatment but not specifically VV or VA-ECMO.

Finally, we aimed to test, if early, compared to late, use of haemoadsorption might be associated with benefits in patients’ outcomes (Table 3). Most patients in the haemoadsorption group were admitted to the ICU within two days of hospitalisation (median 0 [IQR 0–25] hours for the survivors and 0 [0–28] hours for the non-survivors, *p* = 0.5116). After ICU admission, the survivors received haemoadsorption significantly earlier (5 [1-13] days) than the non-survivors (7 [2-14] days, *p* = 0.0104). We dissected the timeframe from ICU admission to the initiation of haemoadsorption into five medically relevant intervals (Table 3). Most patients received haemoadsorption later than 87 h after ICU admission (52.9% of the survivors and 63.9% of the non-survivors) or within the first 24 h (25.3% of the survivors and 17.7% of the non-survivors). The use of haemoadsorption had no significant effect on survival in any of the observed intervals for therapy onset.

**Table 3:** Temporal overview of patient care in survivors and non-survivors in the haemoadsorption group. The p values indicate the statistical impact on survival, eventually when haemoadsorption is deployed within a certain time frame upon ICU admission.

	Survivors (*n* = 261)		Non-survivors (*n* = 768)		
*Patient care timings*,* median (IQR) in days*					*p*
ICU admission to deployment of haemoadsorption	5 (1–13)		7 (2–14)		0.0104
Hospitalisation to deployment of haemoadsorption	7 (2–15)		9 (3–17)		0.0095
*ICU admission to deployment of haemoadsorption*					
≤ 24 h	66	(25.29%)	136	(17.71%)	0.8348
> 24 h – ≤ 48 h	19	(7.28%)	52	(6.77%)	0.5493
> 48 h – ≤ 72 h	13	(4.98%)	41	(5.34%)	0.4317
> 72 h – ≤ 87 h	10	(3.83%)	24	(3.13%)	0.6197
> 87 h	138	(52.87%)	491	(63.93%)	0.6773
Total*	246	(94.25%)	744	(96.88%)	

Data are n (%), unless otherwise stated. The time refers to the period from admission to the treatment clinic to the start of haemoadsorption therapy and does not take into account possible earlier treatment in the event of a transfer. *timing data was not available for all patients. ICU, intensive care unit; IQR, interquartile range; OR, odds ratio; CI, confidence interval.

**Fig. 3:**
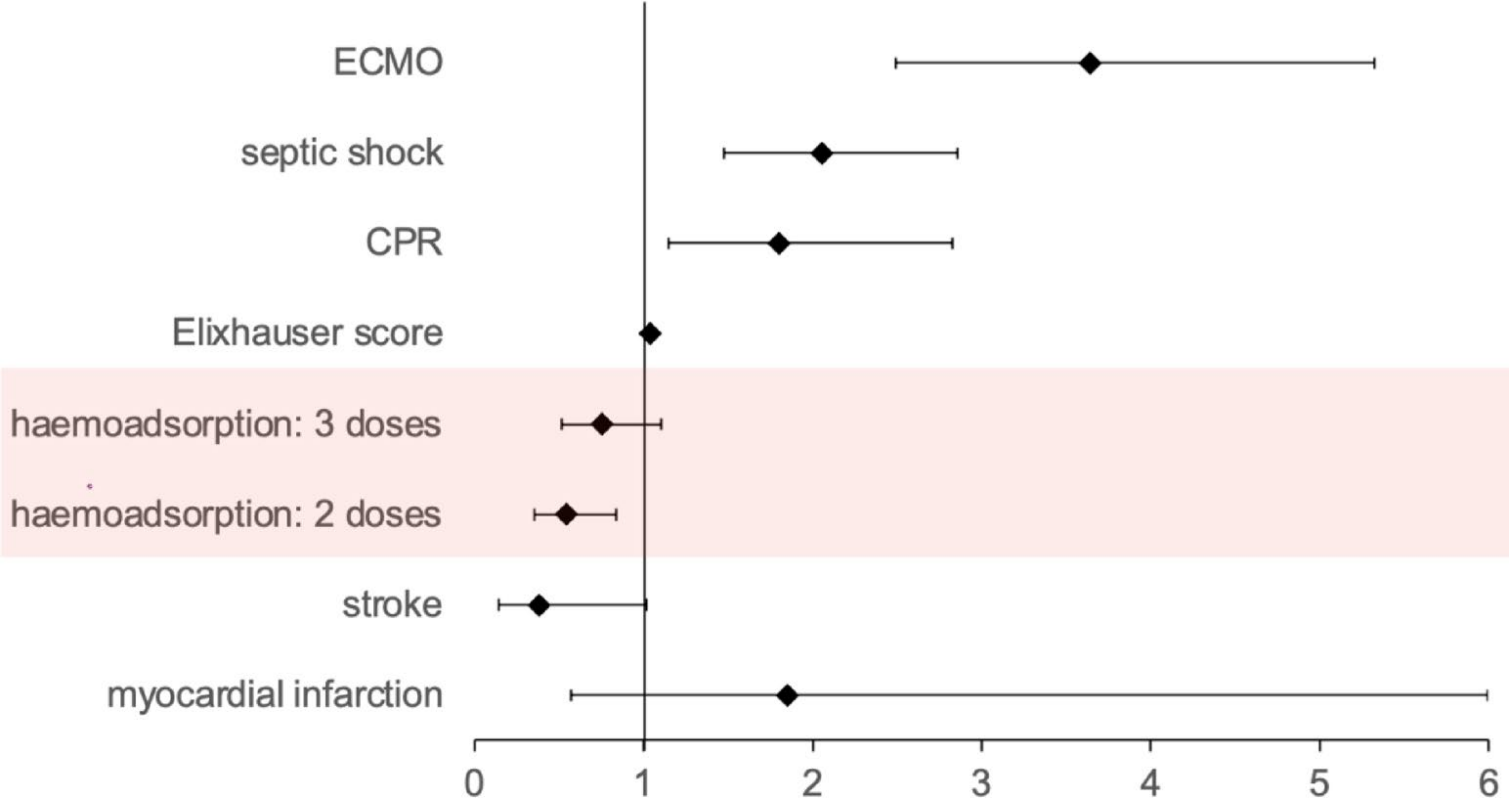
The timing of therapy initiation has no effect on survival in the multiple logistic regression model within the haemoadsorption group. The multiple logistic regression model for the haemoadsorption group included a spline function for the time of therapy initiation after ICU admission to account for the non-linear distribution of patients’ treatment onset, which had no significant impact on mortality. Statistically insignificant parameters that were included in the model with a spline function for timing of therapy initiation after ICU admission due to AIC improvement are three doses versus one dose of haemoadsorption (0.75 [0.51–1.10]), stroke (0.38 [0.14–1.02]), and myocardial infarction (1.85 [0.57–5.99]). ECMO = extracorporeal membrane oxygenation, CPR = cardiopulmonary resuscitation.

In the multiple logistic regression model for the haemoadsorption group, the probability of mortality was dependent on the use of ECMO (3.64 [2.49–5.32]), septic shock (2.06 [1.48–2.86]), CPR (1.80 [1.15–2.83]), and the Elixhauser score (1.04 [1.02–1.06]), but not on the timing of the therapy. Two applications of haemoadsorption significantly increased survival chance compared with one application (0.55 [0.36–0.84]), but this effect is not statistically significant when three cartridges were applied (0.75 [0.51–1.10]). The model was additionally adjusted to stroke and myocardial infarction (Fig. 3).

## Discussion

In our nationwide observational study, we found a significantly higher risk of in-hospital mortality in haemoadsorption patients compared to a matched control group, suggesting that the widespread use of this technique in COVID-19 is not justified. Of note, ECMO support and CPR were also associated with increased mortality as independent variables in the logistic regression model. However, we emphasise that while ECMO and CPR are essential interventions that replace or restore life-sustaining functions, haemoadsorption is an adjunctive therapy whose potential benefits and risks to the patient must be carefully weighed. The use of haemoadsorption therefore requires more nuanced medical and ethical considerations for application in clinical practice.

Few small and non-confirmatory RCTs from Germany have evaluated the use of haemoadsorption with the CytoSorb® device in critically ill COVID-19 patients^22–24^. In patients with vasoplegic shock and renal replacement therapy (*n* = 49), 30-day mortality was not significantly improved by haemoadsorption (74% vs. 58% in the control group, *p* = 0.23). Neither effect on IL-6 levels, catecholamine requirement, nor other clinical endpoints was found in this study^24^. The CYCOV trial included COVID-19 patients supported with VV-ECMO (*n* = 34) and revealed a significantly higher 30-day mortality in the haemoadsorption group (76% vs. 18% in the control group, *p* = 0.0016), however, mortality was assessed only as a secondary endpoint^23^. Another RCT with COVID-19 patients failed to demonstrate a significant mortality benefit (*n* = 24; 28-day mortality 58% in the intervention group vs. 67% in the control group, *p* = 1.0). Of note, in this trial, increased IL-6 levels at or above 500 ng/L were among the inclusion criteria^22^. Despite a previous promising report of CytoSorb® therapy during VV-ECMO in COVID-19 patients (*n* = 8)^18^, no significant reduction of IL-6 plasma levels could be observed^23,24^.

In our cohort, in-hospital mortality was significantly higher in patients who received haemoadsorption compared to those treated without. These findings confirm concerns regarding the safety of uncritical use of this technique in clinical routine for severely ill COVID-19 patients. Recent studies challenge the relevance of hypercytokinemia in critical COVID-19^14,15^. A previous single-centre study shows significantly lower levels of IL-6, IL-8, and tumor necrosis factor (TNF) in patients with COVID-19-associated ARDS (*n* = 62) compared to septic shock patients with (*n* = 51) and without (*n* = 15) ARDS and a mixed picture when comparing COVID-19 to trauma (*n* = 62) and out-of-hospital cardiac arrest (*n* = 30)^15^. In a meta-analysis of multiple published cohorts, pooled mean IL-6 plasma levels for COVID-19 patients (*n* = 1,245) were significantly lower compared to other critical conditions, including chimeric antigen receptor (CAR)-T cell-induced cytokine release syndrome (100-fold difference, *n* = 72), hyperinflammatory ARDS (50-fold difference, *n* = 868), sepsis (30-fold difference, *n* = 5,320), and hypoinflammatory ARDS (5-fold difference, *n* = 1,899)^14^. A similar trend was observed for IL-8, TNF and other pro-inflammatory cytokines, while acute phase reactants were elevated, suggesting a hypoimmune state with virus-mediated tissue damage as the cause of critical COVID-19^14^. Based on these concerns and the findings from the above mentioned RCTs, the rationale and the safety for the routine use of haemoadsorption should be critically considered. We therefore suggest limiting the use of haemoadsorption in COVID-19 to carefully designed clinical trials.

Excessive release of cytokines is a hallmark of sepsis and ARDS independent from COVID-19^14^. Recent randomised evidence suggested that haemoadsorption may be useful as a therapeutic approach in sepsis and septic shock^31^. Based on the growing body of literature^1^, we hypothesised that a septic shock may have been the indication for haemoadsorption in our study population rather than critical COVID-19. To account for the interdependency of haemoadsorption and septic shock, we introduced interaction variables in regression modelling. The results confirmed that there was no survival benefit from haemoadsorption in either the overall group or in patients with septic shock. Similarly, a retrospective propensity score matched analysis of septic patients with hypercytokinemia (*n* = 143; mean initial IL-6 levels of 58,000–60,000 ng/L) showed neither difference in IL-6 reduction, haemodynamic stabilisation, nor mortality for patients treated with haemoadsorption^32^. These findings suggest that the use of haemoadsorption requires more stringent patient selection criteria and is not necessarily justified for septic shock patients with COVID-19^3,5^.

Our granular analysis delineates several critical factors related to mortality among patients receiving haemoadsorption that should be considered and further assessed in clinical trials. Advanced age and poor health, as assessed by the Elixhauser score, pose a high risk of death. Haemoadsorption did not provide a survival benefit for critically ill COVID-19 patients who required ECMO, CPR and/or dialysis. Of note, more than half of the CPR patients in our haemoadsorption cohort (54.3%, 108/199) received this treatment after IHCA. Previously, the use of haemoadsorption showed no benefits after extracorporeal CPR in a single-centre RCT (*n* = 41) and was associated with higher mortality for out-of-hospital cardiac arrest patients (*n* = 72) in a propensity-matched single-centre registry study^33,34^. Due to inherent limitations, we cannot determine if the haemoadsorption treatment was related to CPR but nevertheless, our data do not suggest a positive impact of the haemoadsorption therapy on the mortality of COVID-19 patients at any time point after IHCA. While male sex is known to have a higher risk of COVID-19-related ICU admission and death^35,36^, gender was matched and did not play a detrimental role in our study population. The deployment of haemoadsorption correlated with higher numbers of coagulopathies, cardiac arrhythmia, and CPR but with lower numbers of pulmonary embolism and stroke. At the same time, coagulation disorders were associated with an increased risk of death in the control group, which is consistent with numerous studies confirming the cardiovascular manifestations of COVID-19 and their poor prognosis for patients^37^. The COVID-19 anticoagulation guidelines in Germany have been adjusted several times during the pandemic to balance the risk of thrombosis and haemorrhage^38^. Within our study, the patient cohort experienced a spectrum of treatment regimens reflective of an iterative learning process in protocol development, which may have led to an elevated rate of cardiovascular mortality in the control group due to suboptimal anticoagulation targets. In contrast, haemoadsorption therapy was administered under consistent, manufacturer-recommended anticoagulation protocols, potentially attenuating the risk of fatal cardiovascular outcomes. Acute liver failure was detrimental to survival in both groups, although previous case studies on the use of haemoadsorption for this indication in non-COVID-19 patients were positive^39,40^. Further, our data confirm a significant risk for cardiac arrhythmia associated with haemoadsorption therapy, which has also been reported in the RCT with vasoplegic shock patients (12 events in *n* = 10, 47.6% of patients in the haemoadsorption group vs. 3 events in *n* = 3, 11.5% patients in the control group, *p* = 0.0115)^24^. Patients with malignant diseases had a significantly increased risk of death if they were treated with haemoadsorption, which calls for particular caution. Even though a causality cannot be established with the current evidence, the risk factors derived in our study, among others, must be closely monitored and reported in future studies involving haemoadsorption.

Early initiation of haemoadsorption has been suggested for the treatment of septic shock patients^19^. In the retrospective CTC registry-based study (*n* = 100), early (≤ 87 h) use of haemoadsorption was associated with a reduction of the duration of mechanical ventilation (7 [2–26] days in the early treatment group vs. 17 [7–37] days in the late treatment group, *p* = 0.02), less days of ECMO support (13 [8–24] vs. 29 [14–38] days, *p* = 0.021), and shorter ICU stays (17 [10–40] vs. 36 [19–55] days, *p* = 0.002)^20^. However, no significant reduction of 90-day mortality was observed in the early treatment group (18%) compared to the late therapy start (34%)^20^, and the study suffered from serious methodological shortcomings, most importantly the absence of a control group^21^. We did not observe a significant impact on survival by early versus late therapy initiation in the multiple logistic regression model with a spline function controlling for therapy timing. Of note, our haemoadsorption group cannot be directly compared with the CTC patients, as our dataset does neither include laboratory values, respiratory parameters, nor the sequential organ failure assessment (SOFA) score, which are not included in the DESTATIS datasets. Nevertheless, 90-day mortality was remarkably lower among the retrospectively selected haemoadsorption patients on VV-ECMO support from the CTC registry (26%) than the in-hospital mortality in German ICUs (*n* = 357, 81% of all 443 VV-ECMO patients treated with haemoadsorption), suggesting a highly selected patient cohort in the CTC registry limiting the generalisability of the results. Still, the timing of COVID-19 treatment is crucial for immunomodulatory approaches like e.g. the widely accepted anti-IL-6 antibody tocilizumab^41^, and needs to be carefully evaluated also for haemoadsorption.

COVID-19 was shown to manipulate numerous biological pathways and cause a highly individual immune response^42,43^. Attempts to characterise the specificity of the CytoSorb® haemoadsorption device in septic and cardio-operative patients showed very different specificity profiles and demonstrated in some patients the elimination of apolipoprotein A1, serotransferrin, α-1-antitrypsin, immunoglobulins, and other proteins that are key in numerous biological pathways^44^. Many of these proteins are not quantified in routine clinical practice, making it difficult to understand their role for patient outcomes. It is therefore reasonable to assume that the non-specific and still undefined removal of biomolecules from the blood may have adverse effects that could reduce or eliminate the benefits of haemoadsorption in some patient groups. For example, longitudinal biomarker profiling of ECMO patients with and without haemoadsorption (*n* = 22) revealed a reduction of IL-10, that was speculated to exacerbate COVID-19-induced organ damage^8^. Further, clinicians must consider that haemoadsorption has been reported to adsorb various drugs and their active intermediates in vitro and in vivo^9^. Given the advanced age and poor health of non-surviving haemoadsorption patients, the removal of critical drugs such as immunosuppressants, antiepileptics, antiinfectives, and anticoagulants from the patients’ blood can be fatal.

### Limitations

This observational study has limitations. The analyses were conducted with retrospective data, which were provided by the Federal Statistical Office. Due to the institutionally ensured anonymity, no conclusions can be drawn at individual patient level. Even if the sequence of events or treatment indications cannot be determined, we can confirm independent risk factors for mortality by regression modelling. We cannot provide laboratory data on inflammatory marker profiles or essential concomitant medication since they are in most cases not coded for reimbursement. Individual clinical parameters such as oxygen indices or the clinical presence of ARDS were not included in the matching process, as these variables are not coded for reimbursement and are therefore not available in the data set. Likewise, the reconstruction of clinical scoring systems (such as APACHE II or SOFA) using remuneration data alone is methodologically limited. To avoid misinterpretation suggesting the inclusion of clinical parameters (e.g. lab values or blood gas analyses), these metrics were not reported. This represents a limitation with regard to comparability with other studies that include detailed clinical parameters. Long-term survival analysis be conducted based on this dataset. In contrast, the Elixhauser Score a validated measure of disease severity derivable from remuneration records and is validated for hospitalised patients^25,45^. A possible coding-related bias in the Elixhauser score would affect all patient groups in our analysis equally. Furthermore, we assume that any facility-specific bias in our analysis is largely averaged out by the large number of hospitals (204 ICUs) covered in a short period of time. Our analysis does not provide indications and medical rationales for the use of haemoadsorption (e.g. myoglobin removal during rhabdomyolysis)^46^. Further, we cannot evaluate the physician’s assessment of the patient’s state. Specifically in our extremely vulnerable study group, the physician’s decision to use a costly and disputable therapeutic approach may have led to a bias in the selection of those patients who appeared to have the best chance of survival. Notably, the OPS codes for multiple haemoadsorption devices and no conclusion can be drawn regarding the differences in the devices from different manufacturers.

## Conclusion

In summary, our nationwide analysis of all 1029 patients treated with haemoadsorption in German ICUs during the height of the COVID-19 pandemic in 2020 and 2021 demonstrates that haemoadsorption may be potentially associated with increased in-hospital mortality. Previous clinical evidence for the use of haemoadsorption in critical COVID-19 is weak and the scientific rationale does not support its widespread use, but rather strict patient selection. Thus, currently the application of haemoadsorption should be limited to carefully selected patients within high-quality randomized controlled trials assessing meaningful patient-centered outcomes.

## Supplementary Information

Below is the link to the electronic supplementary material.

Supplementary Material 1

## Data Availability

The data that support the findings of this study are available from the Federal Statistical Office, but restrictions apply to the availability of these data, which were used under license for the current study and thus are not publicly available. Data are, however, available from the corresponding author upon reasonable request and with permission of the Federal Statistical Office.
