# Supplementary material for: Critical risks of haemoadsorption for COVID-19 patients and directions for future evaluations: a nationwide propensity score matched cohort study
Source: Sci Rep. 2025 Aug 9;15:29184. doi: 10.1038/s41598-025-13860-0 (PMC12335523; doi:10.1038/s41598-025-13860-0)
Supplement: Supplementary file 1 — Supplementary Material 1 [file 41598_2025_13860_MOESM1_ESM.pdf]

Supplementary material

Secondary diagnosis	ICD10 codes	Elixhauser score
Congestive heart failure	I09.9, I11.0, I13.0, I13.2, I25.5, I42.0, I42.5 I42.9, I43.x, I50.x, P29.0	yes
Hypertension, uncomplicated and hypertension, complicated, summarised as hypertension	I10.x, I11.x–I13.x, I15.x	yes
Chronic pulmonary disease	I27.8, I27.9, J40.x–J47.x, J60.x–J67.x, J68.4, J70.1, J70.3	yes
Chronic liver disease	B18.x, I85.x, I86.4, I98.2, K70.x, K71.1, K71.3–K71.5, K71.7, K72.x–K74.x, K76.0, K76.2–K76.9, Z94.4	yes
Diabetes, uncomplicated and diabetes complicated summarised as diabetes	E10.0, E10.1, E10.9, E11.0, E11.1, E11.9, E12.0, E12.1, E12.9, E13.0, E13.1, E13.9, E14.0, E14.1, E14.9, E10.2–E10.8, E11.2–E11.8, E12.2E12.8, E13.2–E13.8, E14.2–E14.8	yes
Renal disease*	I12.0, I13.1, N18.x, N19.x, N25.0, Z49.0, Z49.2, Z94.0, Z99.2	yes
Lymphoma, metastatic cancer, solid tumour without metastatis, summarised as malignant diseases	C81.x–C85.x, C88.x, C96.x, C90.0, C90.2, C77.x–C80.x, C00.x–C26.x, C30.x–C34.x, C37.x–C41.x, C43.x, C45.x–C58.x, C60.x–C76.x, C97.x	yes
Coagulophathy	D65–D68.x, D69.1, D69.3, D69.6	yes
Obesity	E66.x	yes

*Renal disease includes hypertensive kidney disease, hypertensive heart and kidney disease with renal failure, chronic kidney disease of all stages, unspecified renal failure, preparation for dialysis, other dialysis except extracorporeal dialysis, condition after kidney transplantation, and long-term dependence on dialysis in renal failure. ICD10 = 10th version of the International Statistical Classification of Diseases and Related Health Problems

Supplementary table 1: ICD10 codes of comorbidities. Most comorbidities were included in the calculation of the weighted Elixhauser score, which has been constructed in a separate SAS request.

Secondary diagnosis	ICD10 codes
Acute liver failure	K72.x
Intracranial bleeding	I60.x, I61.x, I62.x
Stroke	I63, I64

Secondary diagnosis	ICD10 codes
Pulmonary embolism	I26.x
Embolism and/or thrombosis	I74.x
Myocardial infarction	I21., I22., I24.
Cardiac arrythmia	I44.1–I44.3, I45.6, I45.9, I47.x–I49.x, R00.0, R00.1, R00.8, T82.1, Z45.0, Z95.0
Circulatory arrest prior to hospitalisation	U69.13
Cerebral edema	G93.6
Anoxic brain damage	G93.1
Acute renal failure	N17.0-N17.99
Septic shock	R57.2

ICD10 = 10th version of the International Statistical Classification of Diseases and Related Health Problems

Supplementary table 2: ICD10 codes used for the request of complications.

Procedure	OPS code
Haemoadsorption	8-821.2
CPR	8-771, 8-772, 8-779
Dialysis	8-853., 8-854., 8-855.
ECMO	8-852.0, 8-852.3, 8-852.6

OPS = operation and procedure code (Operationen- und Prozedurenschlüssel), CPR = cardiopulmonary resuscitation,
ECMO = extracorporeal membrane oxygenation including veno-venous, veno-arterial, and RA-PA ECMO

Supplementary table 3: OPS codes used for the request of medical procedures.
